# Intramuscular immunization of mice with live influenza virus is more immunogenic and offers greater protection than immunization with inactivated virus

**DOI:** 10.1186/1743-422X-8-251

**Published:** 2011-05-21

**Authors:** Katie Harris, Rebecca Ream, Jin Gao, Maryna C Eichelberger

**Affiliations:** 1 Division of Viral Products, Office of Vaccine Review and Research, Center for Biologics Evaluation and Research, Food and Drug Administration, USA

## Abstract

**Background:**

Influenza virus continues to cause significant hospitalization rates in infants and young children. A 2-dose regime of trivalent inactivated vaccine is required to generate protective levels of hemagglutination inhibiting (HAI) antibodies. A vaccine preparation with enhanced immunogenicity is therefore desirable.

**Methods:**

Mice were inoculated intramuscularly (IM) with live and inactivated preparations of A/Wisconsin/67/2005 (H3N2). Serum cytokine levels, hemagglutinin (HA)-specific antibody responses and nucleoprotein (NP)-specific CD8+ T cell responses were compared between vaccinated groups, as well as to responses measured after intranasal infection. The protective efficacy of each vaccine type was compared by measuring virus titers in the lungs and weight loss of mice challenged intranasally with a heterosubtypic virus, A/PR/8/34 (H1N1).

**Results:**

Intramuscular administration of live virus resulted in greater amounts of IFN-α, IL-12 and IFN-γ, HA-specific antibodies, and virus-specific CD8+ T cells, than IM immunization with inactivated virus. These increases corresponded with the live virus vaccinated group having significantly less weight loss and less virus in the lungs on day 7 following challenge with a sublethal dose of a heterosubtypic virus.

**Conclusions:**

Inflammatory cytokines, antibody titers to HA and CD8+ T cell responses were greater to live than inactivated virus delivered IM. These increased responses correlated with greater protection against heterosubtypic virus challenge, suggesting that intramuscular immunization with live influenza virus may be a practical means to increase vaccine immunogenicity and to broaden protection in pediatric populations.

## Background

Influenza-infected infants and children younger than 2 years old are at increased risk for severe respiratory disease, requiring hospitalization [1]. The mortality due to infection is high for newborn infants and children that are immuno-compromised, as well as children with underlying heart or lung disease [2]. Treatment options are limited since many influenza strains are resistant to licensed antivirals [3]. In order to prevent this burden of disease, vaccination against influenza is recommended for all children older than 6 months [4].

Both inactivated and live influenza virus vaccines are available. The live vaccine preparation is licensed for use in 2 - 49 year olds, but because it is administered intranasally (IN), is not recommended for individuals who have a history of wheezing or diagnosis of reactive airway disease. Individuals that cannot receive the live attenuated vaccine due to age, immune status or respiratory disease can be vaccinated intramuscularly (IM) with the inactivated influenza preparation. Two doses are recommended when children are vaccinated for the first time to achieve desirable seroconversion [4,5]. While this regimen offers immunity against influenza, it is often difficult to achieve both doses [6], and therefore influenza vaccine preparations that are more immunogenic and offer greater protection against different strains, are desirable.

Immunogenicity of protein antigens is determined by a plethora of events that follow the activation of pathogen sensors. For influenza, these sensors include TLR7 that binds to single-stranded RNA in the endosome or NOD-like receptors (NLR) and retinoic acid inducible gene I (RIG-I) receptors that bind to early replication intermediates (double-stranded RNA or 5' phosphate) in the cytoplasm (reviewed in [7]). These sensors work in co-operation with one another [8]: for example, proIL-1β expression is induced through the TLR7 pathway but must be activated by cleavage with caspase-1, a component of the inflammasome. This results in innate responses with characteristic signatures, depending on the quantity and quality of these early signals. These early inflammatory mediators influence the type and magnitude of the resultant antigen-specific adaptive response. For example, IL-1β is induced following influenza infection, enhancing priming of CD4+ T cells and induction of IgM [9].

Qualitatively distinct adaptive immune responses have been noted following live and inactivated influenza vaccination [10,11]. These differences include induction of virus-specific CD8+ T cells by live virus vaccines. We hypothesized that discrete early cytokine responses are elicited following live and inactivated influenza virus IM vaccination, supporting distinct adaptive immune responses. To test this idea, we compared cytokine, antibody and CD8+ T cell responses in mice immunized IM with live and inactivated vaccine preparations. In addition, we compared the breadth of protection afforded by each vaccine type by challenging immunized mice IN with a heterosubtypic virus.

## Methods

### Virus preparation

Influenza virus A/Wisconsin/67/05 X161B (A/WI/05), an H3N2 strain used in vaccine manufacture, and mouse-adapted A/PR/8/34 were prepared by inoculation of 10 day old embryonated chicken eggs. Virus was inactivated by exposure to UV light for 30 minutes, or by heating at 60°C for 1 hr. For UV inactivation, 1 ml aliquots of virus were placed in a 12-well tissue culture plate and incubated on ice approximately 2 inches from the UV light source. Infectivity of each preparation was determined by titration in MDCK cell monolayers as previously described [12], with titer expressed as 50% tissue culture infectious dose (TCID_50_) per ml. The original A/WI/05 virus stock contained 10^7 ^TCID_50_/ml. UV and heat treatments reduced this to 10^4 ^and <10^2 ^TCID_50_/ml, respectively. Both live and inactivated preparations were diluted 1:2 in PBS before immunization of mice with 50 μl of inoculum. Each mouse therefore received the same number of particles i.e. the amount present in 2.5 × 10^5 ^50% tissue culture infectious dose units (TCID_50_).

### Study Design

Female BALB/c mice (The Jackson Laboratories, Bar Harbor, Maine) aged 8-10 weeks were used in experiments following protocols approved by the CBER Animal Care and Use Committee in accordance with federal guidelines. Mice were anesthetized by isoflurane inhalation and inoculated with 50 μl of live or UV inactivated A/WI/05 either IM or IN. Three animals per group were sacrificed by CO_2 _asphyxiation on days 1, 2, 3, 7, 14, 21, and 28 post-immunization. The remaining animals were boosted on day 28 and sacrificed on days 2, 7, and 28 post-secondary immunization. Serum was collected by tail-bleed, broncho-alveolar lavage fluid (BAL) was collected by flushing lungs 3-times with 1 ml of PBS-0.1%BSA, and nasal wash (NW) was collected by flushing nasal passages with 0.2 ml PBS-0.1% BSA at each time point. Cellular debris was pelleted and all samples stored at ≤ -20°C.

To evaluate CD8^+ ^T cell responses and protection against disease, immunized mice were challenged IN with a sub-lethal dose of A/PR/8/34 (0.1 LD_50_/mouse) while anesthetized with isoflurane. Mouse weights were measured daily. Mice were euthanized 9 days after challenge to enumerate CD8+ T cells in BAL, whole lung and mediastinal lymph nodes (MLN). Virus titers were determined in lungs collected on day 7 post-challenge.

### Hemagglutination inhibition (HAI) Assay

Non-specific inhibitors were removed from serum by overnight treatment with receptor destroying enzyme (RDE, Accurate Chemical Corp, Westbury, NY) followed by 30 min incubation with packed chicken red blood cells (RBC, CBT Farms, Chestertown, MD). Following centrifugation to remove the RBC, each serum sample was serially diluted in 25 μl PBS and then mixed with an equal volume of PBS containing 4 HAU A/WI/05. After 30 min incubation at room temperature, 50 μl of 0.5% RBC was added and the mixture incubated for 45 minutes before evaluation of agglutination. The titer was recorded as the inverse of the last dilution that inhibited agglutination.

### HA-specific antibody titers measured by ELISA

Recombinant HA of A/WI/05 was purchased from Protein Sciences Corp. (Meriden, CT). Plates (96-well, IMMULON^® ^1B, Thermo Scientific) were coated overnight at 4°C with 25 ng/well HA or left uncoated to account for non-specific antibody binding to the plate. Excess antigen was discarded and the plates blocked during 1 hr incubation at room temperature with 1% BSA Blocking Solution (KPL, Gaithersburg, MD). After washing the plates, 2-fold serial dilutions of samples were added and the plates incubated for 2 hr at room temperature. The plates were then washed and horseradish-peroxidase-conjugated goat anti-mouse Ig (H+L) or individual Ig isotypes IgG1, IgG2a, IgG2b (Southern Biotech, Birmingham, AL) added. After 1 hr incubation the plates were washed and ABTS Peroxidase Substrate (KPL, Gaithersburg, MD) added. Absorbance was read at 450 nm (Victor V, PerkinElmer, Waltham, MA). Titers were assigned as the inverse of the sample dilution in which absorbance was at least 2 times greater in the HA-coated well than non-coated well. To compare the proportion of HA-specific antibodies with a particular isotype, results were documented as the percent of the titer attributed to each isotype, i.e. (titer of each individual isotype/sum of individual titers)x100. Since the sensitivity of each isotype-specific antibody may be different, this calculation provides the means to compare relative amounts of each isotype but does not reflect absolute antibody quantities.

### Cytokine Quantitation

Cytokine analysis was performed on all samples using the Meso Scale Discovery (MSD; Gaithersburg, MD) platform. A multiplex 96-well plate format was used, with simultaneous measurement of IL-1β, IL-2, IL-4, IL-5, mKC, IL-10, IL-12p70, IL-13, IFNγ, and TNFα. All reagents and pre-coated plates were purchased from MSD, and the manufacturer's protocol was followed. Briefly, all reagents were brought to room temperature prior to use, and all incubations performed at room temperature with shaking. Prior to the addition of samples and calibrators the plates were incubated with assay diluent for 30 minutes. Samples and calibrators were added and incubated for 2 hours. After washing the plates three times with PBS-0.05% Tween-20, detection antibody was added and the plates incubated for an additional 2 hours. A final wash was performed, followed by the addition of read buffer. The plates were read using the MSD Sector Imager 2400. Cytokine concentrations were determined using a curve fit model with software provided with the instrument.

### Enumeration of virus-specific CD8+ T cells

BAL, MLN and lungs from 5 mice in each group were pooled. Cells in the BAL were pelleted and resuspended in 5 mL PBS/0.1% BSA. The cell suspension was transferred to a 100 mm non-tissue culture treated petri-dish for macrophage depletion by plastic adherence for 90 minutes at 37°C. Following incubation, the cells that remained in suspension were removed and pelleted prior to treatment with RBC lysing buffer (Sigma, St Louis, MO). To prepare a lymphocyte suspension from lungs, the tissue was cut into small fragments, then forced through a metal strainer. Large particulates were removed by passing the suspension over a 70 μm nylon cell strainer, and the cell suspension was pelleted prior to RBC lysis. MLN were gently homogenized and the cell suspension pelleted. Lymphocytes were counted in each preparation and approximately 1 × 10^6 ^cells were used for immunostaining with an antibody mixture specific for cell surface markers of lymphocyte subsets or isotype controls. The antibody mixture contained anti-CD3-APC-Cy7, anti-CD4-PE, anti-CD8-PerCP Cy5.5, B220-PE-Cy7, and NK1.1-FITC. The antibody mixture was only incubated with the cells after a 10 minute incubation at room temperature with NP_147-155_-MHC class I Pentamer TYQRTRALV-H2K^d ^(ProImmune, Oxford UK). Cells were finally resuspended in 0.5 ml PBS-0.1% BSA and events collected on a FACSCanto (Becton-Dickinson, Franklin Lakes, NJ). Results were analyzed using FloJo 7.2.4.

### Statistical Analysis

Student's t test was used to compare amounts of cytokines present in serum, BAL and nasal wash at each time point within each vaccination group. ANOVA, with Bonferroni post-test, was used to compare differences between groups. Statistical significance was inferred when p ≤ 0.05.

## Results

### Live virus vaccination IM induces more robust innate cytokine responses

**Figure 1:**
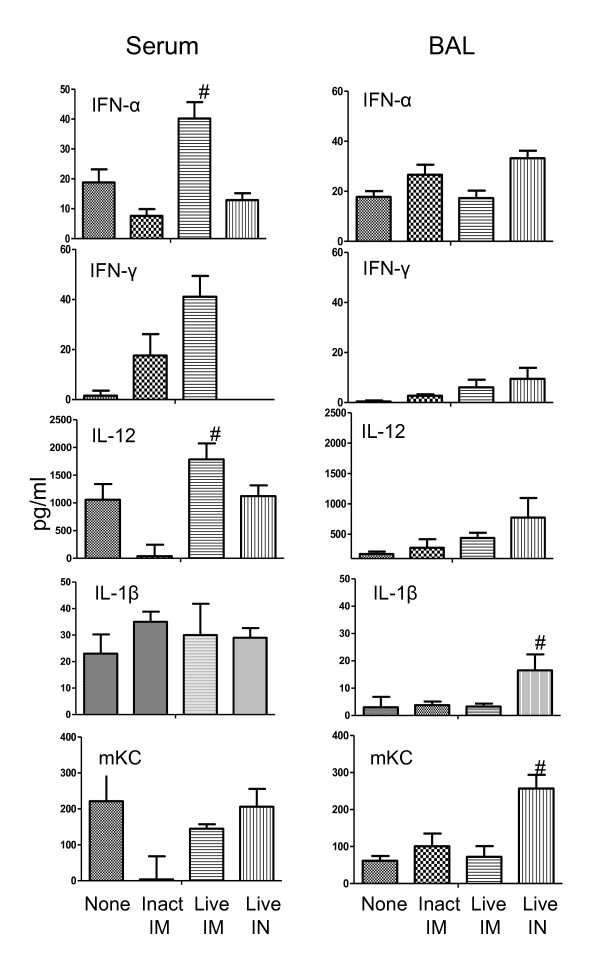
**Average cytokine concentration (pg/ml) in serum and BAL before (none), 1 day after IM vaccination with inactivated and live A/WI/05 and 1 day after IN inoculation with live virus**. The results shown are the average concentration measured in sera or BAL from 3 mice in each group. Significant difference with non-immunized group denoted with *, significant difference with UV-inactivated IM immunized group denoted with # (ANOVA with Bonferroni post-test, significantly different when p ≤ 0.05).

To compare innate responses following IM vaccination with live and UV-inactivated influenza virus A/WI/05, the concentrations of cytokines were measured in serum and BAL collected at various times after one vaccine dose (3 animals per time point in each group). The kinetics of these cytokine responses were also compared with cytokine levels after infection (intranasal) with live A/WI/05. The sample (serum or BAL) in which cytokine responses were measured reflected the site of inoculation - increased amounts of inflammatory cytokines were measured in BAL following IN infection with live virus, whereas these cytokines were increased in serum after IM vaccination (Figure 1). The amount of serum IFN-α, IFN-γ and IL-12 was significantly greater after IM vaccination with live virus compared to UV-inactivated virus (p < 0.05, Figure 1). In contrast to IM vaccination with either live or inactivated virus, elevated levels of mKC, a homolog of the neutrophil chemoattractant IL-8, was measured in BAL after IN infection (Figure 1). Similar results were obtained in a repeat experiment.

**Figure 2:**
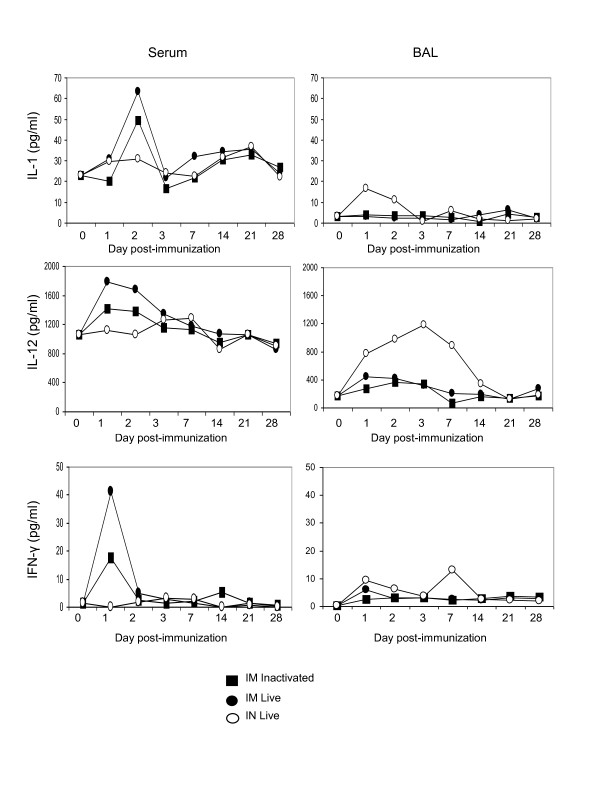
**Kinetics of cytokines present in serum and BAL following primary immunization with live or inactivated A/WI/05 delivered IM or live virus delivered IN**. Mice were immunized and cytokines measured in sera of individual mice using multiplex MSD analysis. Mean IL-1β, IL-12, and IFN-γ concentrations for each group (n = 3) are shown before (day 0) and after immunization.

The route of inoculation also impacted the kinetics of the response - serum IL-1β peaked on day 2 following IM inoculation but peaked on day 1 in BAL after IN infection; IL-12 peaked on day 1 following IM inoculation but on day 3 after IN infection (Figure 2). There were 2 waves of IFN-γ observed in BAL after IN infection with live virus - in the early phase, peak levels were measured 1 day post-infection (p.i.), and in the later phase, IFN-γ peaked on day 7 p.i. (Figure 2). The cytokine profile at early time points after vaccination reflects an innate response, with cells such as natural killer cells as a possible source of these cytokines, whereas the later phase of the response or following boosting to obtain a recall response, is indicative of cytokines secreted by activated virus-specific T cells.

### HA-specific titers are greatest following IM vaccination with live virus

To evaluate differences in the adaptive immune responses to live and inactivated vaccines, we compared HA-specific antibody titers in serum and BAL. IM immunization with both live and inactivated virus resulted in HA-specific IgG responses measured by ELISA on day 28, with greater titers measured after both primary and secondary immunization with live virus (Table 1). This was most notable in BAL where IM immunization with live virus resulted in 4-fold greater HA-specific IgG titers than inactivated virus. The HA-specific IgG titer in BAL after live virus IM vaccination was similar to the titer in BAL of animals infected IN with live virus even though the titer measured in serum of infected mice was not as robust. IgA was not included in this assessment because the assay lacked sufficient sensitivity to quantify this isotype in BAL.

**Table 1:** HA-specific antibody titers measured by ELISA (Total IgG) or HAI in pooled sera and BAL of immunized mice

			IM vaccine type		IN infection
				
Test	Sample	Day post vaccination	Inactivated	Live	Live
**ELISA (Total IgG)**	Serum	0	<25	<25	<25
		7	<25	<25	<25
		28	400	800	100
		7 days post-boost	800	1600	400
		28 days post-boost	800	1600	800
					
	BAL	0	<2	<2	<2
		7	<2	<2	<2
		28	16	64	32
		7 days post-boost	32	64	64
		28 days post-boost	16	64	64
					
**HAI**	Serum	0	<4	<4	<4
		7	16	16	<4
		28	64	256	256
		7 days post-boost	128	256	128
		28 days post-boost	128	512	128

Hemagglutination inhibition (HAI) titers, a measure of functional activity of HA-specific antibodies, were also measured for serum samples (Table 1). The HAI titers after a single or second vaccine dose of live virus delivered IM were 4-fold greater than the inactivated virus-vaccinated group. Serum HAI titers increased following a second IM dose of either live or inactivated virus, but were not boosted when mice previously infected were rechallenged with live virus.

### Qualities of the adaptive response following IM vaccination with live and UV-inactivated virus are distinct

Protection against disease is often dependent on the type of response and not just overall quantity. Others have demonstrated that the quality of the influenza-specific T cell response induced in mediastinal lymph nodes (MLN) draining the lungs of C57BL/6 mice is dependent on the type of immunogen, with Th1 type cytokine IFN-γ secreted in response to live virus and Th2 type cytokine, IL-4, secreted in response to inactivated virus [13], resulting in the induction of greater numbers of IgG2a and IgG1 antibody secreting cells respectively [14]. We therefore examined the type of response by determining the ratio of each isotype contributing to the HA-specific antibody titer, and examined increases in cytokines representative of Th1 and Th2-type responses.

To compare the relative amounts of IgG isotypes, ELISA HA-specific titers were measured using isotype-specific secondary reagents. The titers of HA-specific IgG1, IgG2a and IgG2b were presented as a simple ratio, or as a percent of the summative titer. For example, a pool of sera from mice boosted IM 28 days earlier with live virus had an IgG1:IgG2a:IgG2b ratio of 50:3200:800 or 1%, 79% and 20%, respectively. IgG2a predominated in serum after IM immunization, irrespective of whether the virus was live or inactivated (Table 2). Unexpectedly, HA-specific antibodies of IgG1 isotype that is characteristic of Th2-type responses were present in greater proportion in BAL after IM immunization with live virus (44%), than inactivated virus (3%). The proportion of IgG1, IgG2a and IgG2b in BAL of live virus-vaccinated mice was the same as infected mice, suggesting this form of antigen induced T cell help that is distinct from that induced by non-replicating antigen.

**Table 2:** Proportion of HA-specific antibodies with specific isotypes in pooled sera and BAL measured by ELISA

		Percent of titer attribute to		
		
Sample	Inoculation route/virus form	IgG1	IgG2a	IgG2b
Serum	IM inactivated	1	88	11
	IM live	1	79	20
	IN live	8	61	31
				
BAL	IM inactivated	3	77	20
	IM live	44	44	12
	IN live	44	44	12

**Figure 3:**
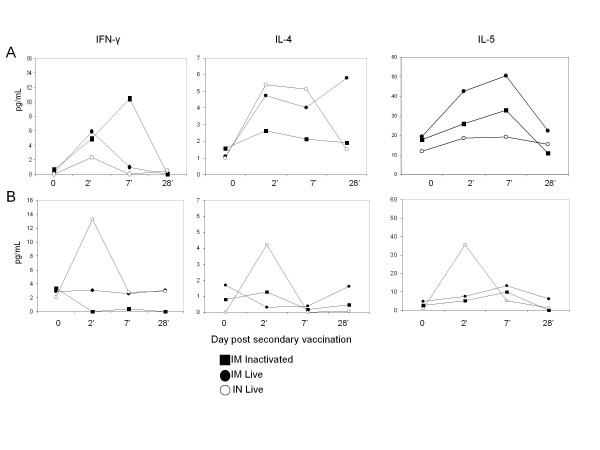
**Kinetics of cytokines present in (A) serum and (B) BAL following secondary IM immunization with live or inactivated A/WI/05, or after a secondary dose of live virus delivered IN**. The concentration of cytokine was measured in samples collected from individual mice using multiplex MSD analysis. Mean values of IFN-γ, IL-4 and IL-5 in sera and BAL of 3 mice per group are shown for each time point following the boosting dose.

Switching to IgG1 and IgG2b isotypes is facilitated by Th2-type cytokines, IL-4 and IL-5, respectively, while Th1-type cytokines support switching to IgG2a. To determine whether the cytokines that support IgG2a, IgG1, as well as IgG2b responses were present following vaccination with live virus, we measured Th1 (IL-2 and IFN-γ) and Th2 (IL-4 and IL-5)-type cytokines in serum and BAL on days 2 and 7 after a second dose of vaccine, when antigen-specific memory T cells are likely to be activated. No increase in IL-2 was detected in serum or BAL at any time point (results not shown). As after primary immunization, increased concentrations of cytokines were evident in the serum, not BAL of mice vaccinated IM. The amount of Th1 type cytokine, IFN-γ, but not Th2-type cytokines IL-4 and IL-5, was increased on day 7 after a boosting dose with inactivated virus. In contrast, IL-4 and IL-5 concentrations were increased in the sera of mice immunized IM with live virus (Figure 3).

Overall, the cytokines measured in serum following a second vaccine dose support the antibody isotypes identified: IM immunization with inactivated virus resulted in increased serum IFN-γ, supporting an IgG2a response, while IM immunization with live virus resulted in increased amounts of IL-4 and IL-5, supporting IgG1 and IgG2b responses, respectively.

### NP-specific CD8^+ ^T cells are present after immunization with live virus

Robust CD8+ T cell responses are measured after IN infection [15]. To establish whether this type of cell-mediated response is elicited following IM vaccination, the number of virus-specific CD8+ T cells was measured by staining lymphocytes from whole lung, BAL and mediastinal lymph nodes (MLN) with NP_147-155_-peptide-loaded H-2K^d ^pentamer in conjunction with antibodies specific for CD8. This peptide is an immunodominant influenza epitope in BALB/c mice [16]. To facilitate the quantitation of these memory cells, mice were challenged with the heterosubtypic A/PR/8/34 (H1N1) virus 28 days after IM vaccination. The sequence of NP_147-155 _is identical in the immunizing (A/WI/05) and challenge (A/PR/8/34) viruses. A control group of mice that had previously been infected with A/WI/05 was used as control. Cells and tissue were obtained 9 days after heterosubtypic challenge and the single cell preparations stained and evaluated by flow cytometry. Table 3 shows the percent of NP_147-155_-specific CD8^+ ^T cells in MLN, lung and BAL of mice immunized IM with live virus. This percent was greater than the proportion of antigen-specific CD8+ T cells activated in response to IM UV-inactivated virus (approximately 3 vs 2% in MLN, 48 vs 27% in lung, 64 vs 33% in BAL). The number of NP-specific CD8+ T cells calculated (percent × total number of cells in each homogenate) showed that IM vaccination with live virus and IN infection resulted in a similar percentage as well as a similar number of antigen-specific CD8+ T cells, suggesting IM vaccination with live virus is an effective means to induce cell-mediated immunity without replication of virus in the lung. The percent as well as number of antigen-specific CD8+ T cells in the UV-inactivated group was greater than the group that had not been vaccinated. Since residual infectious virus in this UV-treated vaccine preparation may account for the induction of some CD8+ T cells, mice were also vaccinated with heat-inactivated virus. The numbers of NP-specific CD8+ T cells recalled into MLN, lung and BAL of mice infected with this latter preparation were minimal in comparison to the non-vaccinated group, if present at all (Table 3). The experiment was repeated with similar results.

**Table 3:** Percent and number of NP-specific CD8+ T cells in pooled MLN, lung and BAL samples after challenge of immunized mice with A/PR/8/34

	Previous exposure to viral antigens				
	
	none	Live virus IN	live virus IM	UV-inact virus IM	heat-inact virus IM
**Percent NP_147-155_-specific CD8+ T cells**					
MLN	0.2	4.2	3.0	1.7	0.3
Lung	9.6	52.0	47.9	26.9	22.0
BAL	15.5	60.2	63.5	32.5	25.6
					
**Number of NP_147-155_-specific CD8+ T cells per mouse**					
MLN	431	10,028	6,296	5,608	1,451
Lung	60,597	231,304	183,918	113,603	62,890
BAL	14,940	23,427	44,129	25,473	13,612

### IM immunization with live virus provides greater protection against heterosubtypic virus challenge than vaccination with inactivated virus

**Figure 4:**
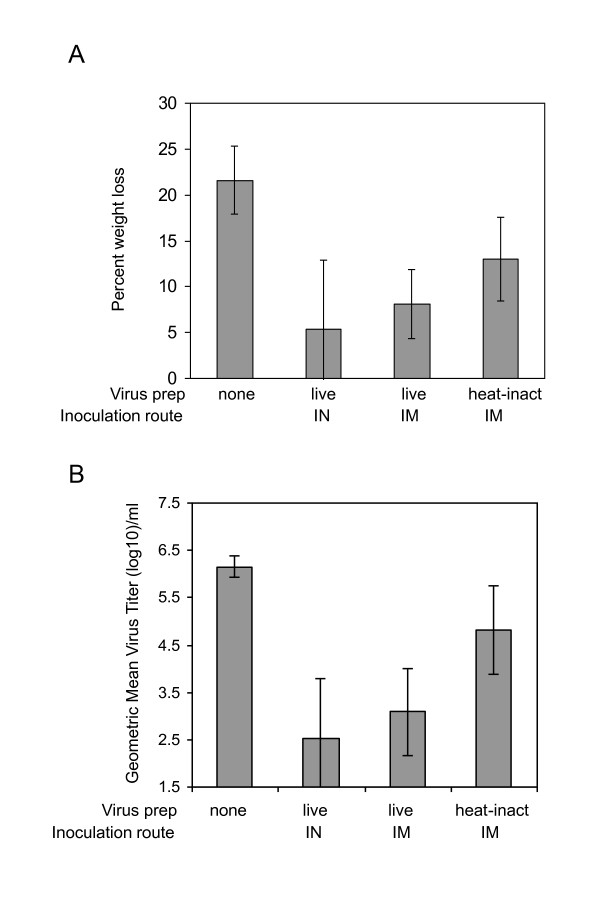
**Protection of mice against heterosubtypic challenge**. Percent weight loss (A) and lung virus titers (B) were measured in A/WI/05 (H3N2)-immunized mice, 7 days after infection with A/PR/8/34 (H1N1). Groups of mice were vaccinated IM with live and heat-treated whole virus. Control groups included mice without prior vaccination and a group of mice infected IN with live A/WI/05.

To determine whether induction of antigen-specific CD8+ T cells in mice vaccinated IM with live virus translated into greater protection against disease, mice were challenged with a sub-lethal dose of A/PR/8/34 and animal weight and virus titers in lungs were measured 7 days post challenge. All immunized groups showed some protection against weight loss and virus replication (p < 0.05 when each group compared to non-vaccinated group), however, mice immunized IM with live virus had significantly less weight loss (p = 0.03) and lower virus titers (p = 0.03) than mice immunized with inactivated virus (Figure 4). IM vaccination with live virus provided a similar level of protection as previous A/WI/05 infection of the respiratory tract (differences in weight loss and lung virus titers were not significant).

## Discussion

Clinical studies show that both live, attenuated and inactivated, split influenza vaccines are safe and effective, although there are instances when greater protection against disease has been observed in young children vaccinated with live, rather than inactivated vaccines [17,18]. This is likely due to differences in the immune mechanisms that contribute to protection: inactivated vaccines induce robust antibody responses [19] that prevent infection or reduce spread of the virus, while live virus vaccines induce cellular responses [20] that probably contribute to protection through secretion of anti-viral cytokines or direct killing of infected cells. Unfortunately the live, attenuated vaccine is administered intranasally, and is therefore contraindicated for individuals with asthma or other respiratory diseases, excluding many children from the possible benefit of cell-mediated immunity. In this report we show differences in the quality and quantity of immune responses to live and inactivated influenza virus administered IM, and demonstrate that as for live virus administered intranasally, IM vaccination with live virus induces robust antibody as well as CD8+ T cell responses, thereby providing protection against challenge with a heterosubtypic virus.

Comparison of cytokine and antibody profiles in sera of mice immunized IM with live and UV-treated virus preparations showed quantitative and qualitative differences, with greater amounts of acute inflammatory cytokines correlating with an increased antibody response following exposure to live virus preparation. Since we used a UV-inactivated virus preparation that retained some live virus, it is possible that even greater differences would have been observed in the complete absence of live virus.

The cytokines measured in this study are induced as a result of signaling through TLR7, RIG-I, and inflammasomes [8]. IL-12 and IFN-γ are usually TLR7-driven, and since virus replication is not required for the interaction of this sensor with its ligand, single-stranded RNA, one would expect both cytokines to be induced following exposure to live and inactivated virus. However, significantly greater amounts of IL-12 and IFN- γ were present 1 day after IM vaccination with live virus than UV-treated virus, even though the latter treatment did not completely inactivate the virus. This may reflect differences in uptake of virions into cells or synergism with other responses that are replication-dependent, for example, an effect of IFN-α that is induced following immunization with the live virus preparation only. This latter idea is in accord with work that demonstrates the ligand for RIG-I is dsRNA [21], and therefore this signal is replication-dependent. IFN-α may contribute to the enhanced immune response in direct as well as indirect means. For example, others have demonstrated synergism between IFN-α and IL-12 [22], and IFN-α is known to increase immunogenicity by acting on B cells to induce early antibody responses [23-25]. It is therefore not surprising that the magnitude of the antibody response following IM immunization with live virus was greater than UV-inactivated virus.

Both live and UV-inactivated virus preparations induced Th1-type responses after the first vaccination, resulting in expected IgG2a HA-specific antibodies. Unexpectedly, IgG1 (IL-4-dependent) and some IgG2b (IL-5 driven) HA-specific antibodies that are typically associated with Th2-type responses were amplified after a second dose of live but not inactivated vaccine. The live virus preparation did indeed result in increased concentrations of IL-4 and IL-5 in serum that could explain these responses.

Our results show that the most robust serum antibody response - measured as either total HA-specific IgG or HAI titers - is generated after IM immunization with live virus. Intranasal inoculation with live virus resulted in similar HAI titers after one dose, but unlike IM vaccination with live virus, HAI titers were not boosted after a second IN exposure to live virus. Priming at the mucosa and in the periphery certainly results in distinct responses that are often important for establishing protection at the appropriate site of infection [26]; IN immunization provides the advantage of inducing local IgA and memory T cell responses that contribute to protection against infection of the upper respiratory tract, whereas IgG in the circulation provides effective protection against influenza infection in the lower respiratory tract of mice [27]. While IM vaccination with live virus probably does not induce local antigen-specific IgA, robust serum HAI titers would contribute to vaccine-induced protection of the lung.

The proposal to administer a live vaccine parenterally is not a new idea - it has long been established that the live virus vaccine for measles, mumps and rubella (MMR) administered intradermally, is an effective pediatric vaccine. This vaccine has excellent immunogenicity when a single dose is delivered at 12 months of age, although a second dose is recommended, to provide a boost to the small percent of recipients (5%) that do not respond to one or more of the antigens after the first dose. Vaccine efficacy is robust and has eliminated endemic measles transmission in the United States [28]. Increasing influenza vaccine immunogenicity by changing the form that is delivered intramuscularly may provide an important increase in effectiveness in the pediatric population.

Unfortunately, maternal antibodies inhibit responses to live virus MMR vaccine delivered parenterally [29]. This is one reason the MMR vaccine is delivered at 1 year of age. Even though we demonstrate boosting of the response to a second dose of live virus delivered IM, careful studies need to be completed in order to establish whether the immune response to influenza can be primed in the presence of influenza-specific antibodies, whether of maternal origin or due to prior vaccination or infection. This will determine whether parenteral immunization with live virus is likely to be immunogenic in seropositive children and adults.

In summary, our results show that live virus is more immunogenic than inactivated virus when delivered intramuscularly. The increased antibody response corresponds with induction of greater amounts of inflammatory cytokines early after primary immunization. This form of antigen allows for activation of antigen-specific cytolytic CD8+ T cells that are the primary means of clearing influenza A viruses that do not have the same HA and NA subtype. Indeed, IM vaccination with live virus of H3N2 subtype induced large numbers of NP-specific CD8+ T cells and offered significantly more protection against heterosubtypic H1N1 virus challenge. Asthmatics or other individuals with respiratory ailments are excluded from receiving live, attenuated influenza vaccines, therefore, intramuscular delivery of this vaccine type may be a useful strategy to increase immunogenicity and efficacy in population groups that are most at risk of disease.

## Abbreviations

BAL: bronchoalveolar lavage fluid; RBC: red blood cells; HA: hemagglutinin; HAI: hemagglutination inhibition; IM: intra-muscular; IN: intra-nasal; MMR: measles, mumps, and rubella; NLR: NOD-like receptors; NW: nasal wash; RIG-I: retinoic acid inducible gene I; TCID_50_: 50% tissue culture infectious dose.

## Competing interests

The authors declare that they have no competing interests.

## Authors' contributions

MCE designed and supervised experiments. KH, BR and JG performed experiments and analyzed results. MCE and KH wrote the manuscript, BR and JG contributed to manuscript revisions. All authors have read and approved the final manuscript.
